# Effects of time delay and space on herbivore dynamics: linking inducible defenses of plants to herbivore outbreak

**DOI:** 10.1038/srep11246

**Published:** 2015-06-18

**Authors:** Gui-Quan Sun, Su-Lan Wang, Qian Ren, Zhen Jin, Yong-Ping Wu

**Affiliations:** 1 Complex Systems Research Center, Shanxi University, Taiyuan, Shanxi 030006, P.R. China; 2 Department of Children’s Medical Laboratory Diagnosis Center, Qilu Children’s Hospital of Shandong University, Jinan 250022, P.R. China; 3 College of Physics Science and Technology, Yangzhou University, Yangzhou, Jiangsu Province, 225002, P.R. China; 4 School of Mathematical Sciences, Fudan University, Shanghai 200433, P.R. China

## Abstract

Empirical results indicate that inducible defenses of plants have effects on herbivore populations. However, little is known about how inducible defenses of plants have influences on herbivore outbreak when space effect is considered. To reveal the relationship between inducible defenses and herbivore outbreak, we present a mathematical model to describe the interaction of them. It was found that time delay plays dual effects in the persistence of herbivore populations: (i) large value of time delay may be associated with small density of herbivore populations, and thus causes the populations to run a higher risk of extinction; (ii) moderate value of time delay is beneficial for maintaining herbivore density in a determined range which may promote the persistence of herbivore populations. Additionally, we revealed that interaction of time delay and space promotes the growth of average density of herbivore populations during their outbreak period which implied that time delay may drive the resilience of herbivore populations. Our findings highlight the close relationship between inducible defenses of plants and herbivore outbreak.

Herbivores are diverse, ranging in size from microscopic zooplankton to the largest of land vertebrates from the point of view of taxonomy and ecology^1^. By feeding on different plant parts or materials, herbivores can affect plant growth, transfers of nutrients to the soil surface, and habitat and resource conditions for other organisms. In many plants, particularly trees, damages or stresses by herbivores populations can result in changes in the chemical, physical or other aspects of leafs, which are called as “inducible defenses”^2^,^3^,^4^,^5^,^6^. Both theoretical and experimental studies have shown that inducible defences affect stability and persistence of herbivore populations^7^,^8^,^9^,^10^,^11^,^12^,^13^,^14^,^15^,^16^.

Empirical findings suggested that populations of many herbivorous insects exhibit outbreak, in which short-lived peaks of high density and lots of fallen leaves alternate with long periods of low density^17^,^18^,^19^,^20^,^21^. As a result, the mechanisms on herbivore outbreak have been attracted considerable attention by ecologists and other experts in the relevant research area. The existing work revealed that interactions with enemies^22^,^23^,^24^, physiological stress^2^,^9^,^11^,^25^, the case that herbivore populations’s parents and grandparents experienced in preceding generations^26^, environmental forcing^27^ and limited resource^28^,^29^ may be the significant factors for herbivore outbreak. Although some previous works link inducible defenses to population-level effects on herbivore^2^,^11^,^25^, internal connections of inducible defenses and herbivore outbreak are far from being well understood. Especially, two main questions need to be well addressed: (1) Can inducible defenses of plants induce herbivore outbreak when space is considered? (2) How do inducible defenses affect the persistence of herbivore populations in different aspects during their outbreak period?

It is difficult to characterize the relationship between inducible defenses and herbivore outbreak empirically due to that long time series of the density of both plant and herbivore is needed. It may provide useful information by constructing mathematical models to explain the phenomenon observed in the real world. Edelstein-Keshet posed a model to show how changes in plant quality have influence on herbivore populations^30^. Clark and Harvell used dynamic-optimization models to estimate the relative fitness consequences of inducible versus constitutive defenses strategies and found that inducible defenses played a more important role^31^. Abbott and Dwyer showed that outbreaking insects may be induced by a food limitation in the herbivore and defoliation and intraspecific competition in the host plant^28^. Anderson *et al.* presented a mathematical model on herbivore competition mediated by inducible changes in plant quality and obtained several types of competition outcomes^32^. Most studies to date only consider the evolution in time^2^,^7^,^9^. Nevertheless, it has been observed in the literature that spatial effects on plant and herbivore had been generally overlooked despite its potential ecological reality and intrinsic theoretical interest. In our paper, we will investigate the plant-herbivore interactions with time delay (it arises between herbivore damage and deployment of inducible defenses) and spatial diffusion and aim to link inducible defences to herbivore outbreak.

## Results

Since overall data of herbivore is not available, we may not find out the intrinsic mechanisms on herbivore outbreak empirically. Instead, we are aim to use a simple model to reflect the interactions between inducible defenses and herbivore populations (see Method section).

Our analysis is to link inducible defenses and herbivore outbreak by three steps. Firstly, we obtain the conditions on critical value of time delay for herbivore outbreak analytically. Secondly, we revealed dual effects of time delay on herbivore outbreak: on the one hand, large value of time delay brings about herbivore density to be zero which implied that time delay may be harmful to survival of herbivore; on the other hand, moderate value of time delay promotes the persistence of herbivore during the stage of herbivore outbreak. Finally, we display that joint forces of time delay and space boost the growth of average density of herbivore populations.

### Critical value of time delay for herbivore outbreak

Based on mathematical analysis, one can find the critical value of time delay to ensure the outbreak of herbivore populations (see Method section). The smallest critical value of time delay has the following form:


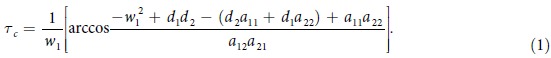


**Figure 1:**
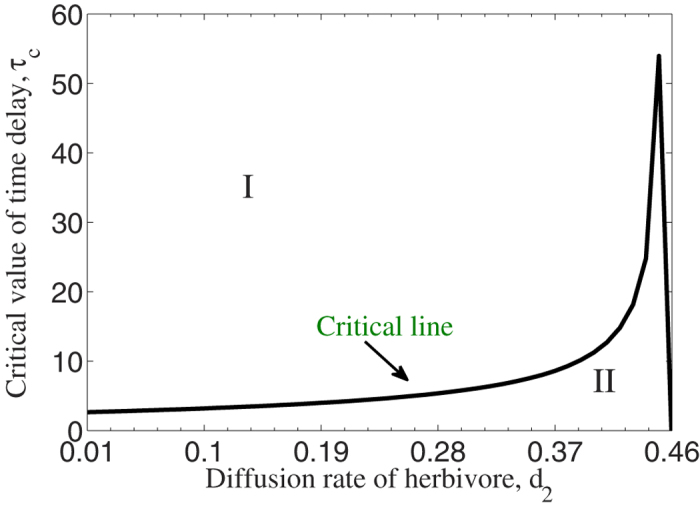
Critical value of time delay with respect to diffusion rate of herbivore populations. This figure indicates that critical value of time delay is an increasing function of diffusion coefficient of herbivore populations as *d*_2_ is small. As *d*_2_ further increases, critical value of time delay is a decreasing function of diffusion coefficient of herbivore populations. Region I: Outbreak domain; II: No outbreak domain.

To well see the effect of time delay on herbivore outbreak, critical value of time delay is shown as a function of diffusion coefficient of herbivore populations in Fig. 1. As seen from this figure that, when the moving speed of herbivore populations is small (*d*_2_ < 0.45), larger diffusion rate of herbivore requires larger value of time delay to ensure their outbreak; when the moving speed is large enough, critical value of time delay is a decreasing function of the diffusion rate. Biological speaking, there is a balance between time delay and spatial motion of herbivore populations in the mechanisms on herbivore outbreak. When diffusion rate of herbivore populations is small, herbivore populations will consume more resources as *d*_2_ increases, which needs larger time delay to hold back their growth and thus herbivore will periodic outbreak. When diffusion rate of herbivore populations is large enough, the remained resources are limited which may induce negative growth of herbivore populations. In this case, smaller time delay can lead to herbivore outbreak.

**Figure 2:**
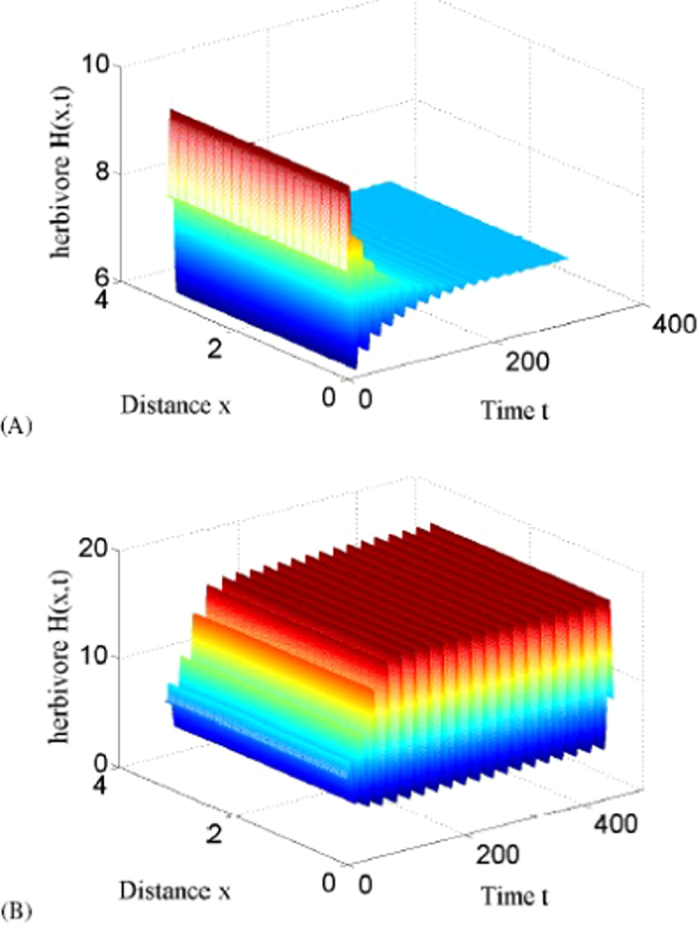
Density of herbivore populations of system (3) with respect to space and time. (**A**) *τ* = 3.2 < *τ*_*c*_; (**B**) *τ* = 4.8 > *τ*_*c*_. Initial conditions are small random perturbation of the positive stationary solution of system (3).

In Fig. 2, we show the herbivore outbreak numerically for fixed parameters sets: *α* = 200, *β* = 1, *δ* = 0.75, *b* = 5, *θ* = 3, *r* = 1, *K* = 10, *m* = 0.01, *d*_1_ = 0.01 and *d*_2_ = 0.25. Under these circumstances, one can find that *τ*_*c*_ ≈ 4.7412. Herbivore populations are considered as a function of space (in one-dimensional space) and time. In Fig. 2(A), the solutions are stable as *τ* = 3.2 < *τ*_*c*_; while in Fig. 2(B), periodic solutions emerge as *τ* = 4.8 > *τ*_*c*_. In other words, time delay induces the outbreak of herbivore populations.

**Figure 3:**
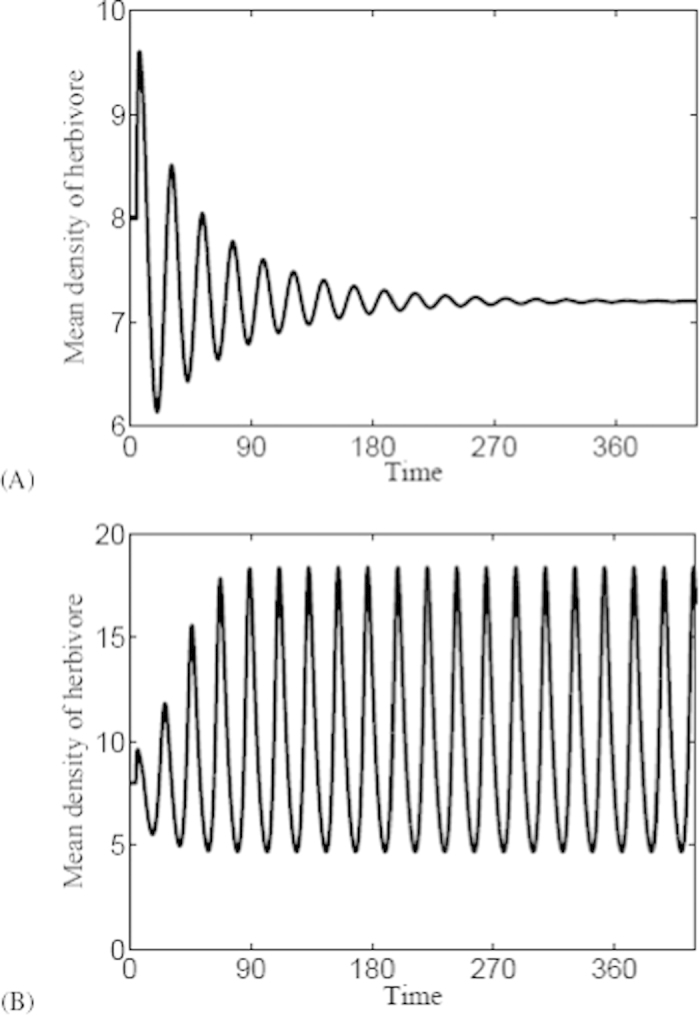
Time series of herbivore populations with 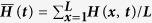 **(L is the space length).** (**A**) Stable solutions with *τ* = 3.2 < *τ*_*c*_; (**B**) Periodic solutions with *τ* = 4.8 > *τ*_*c*_.

In order to better show the outbreak of herbivore populations, time series are shown in Fig. 3. In Fig. 3(A), herbivore populations exhibit a oscillation behavior with decreased amplitude and converge to a constant state with *τ* < *τ*_*c*_. This figure suggests that periodic outbreak of herbivore will not appear with *τ* < *τ*_*c*_. However, when *τ* > *τ*_*c*_, herbivore populations will outbreak with fixed period and amplitude as evolution time is long enough showed in Fig. 3(B).

### Dual effects of time delay on the persistence of herbivore

**Figure 4:**
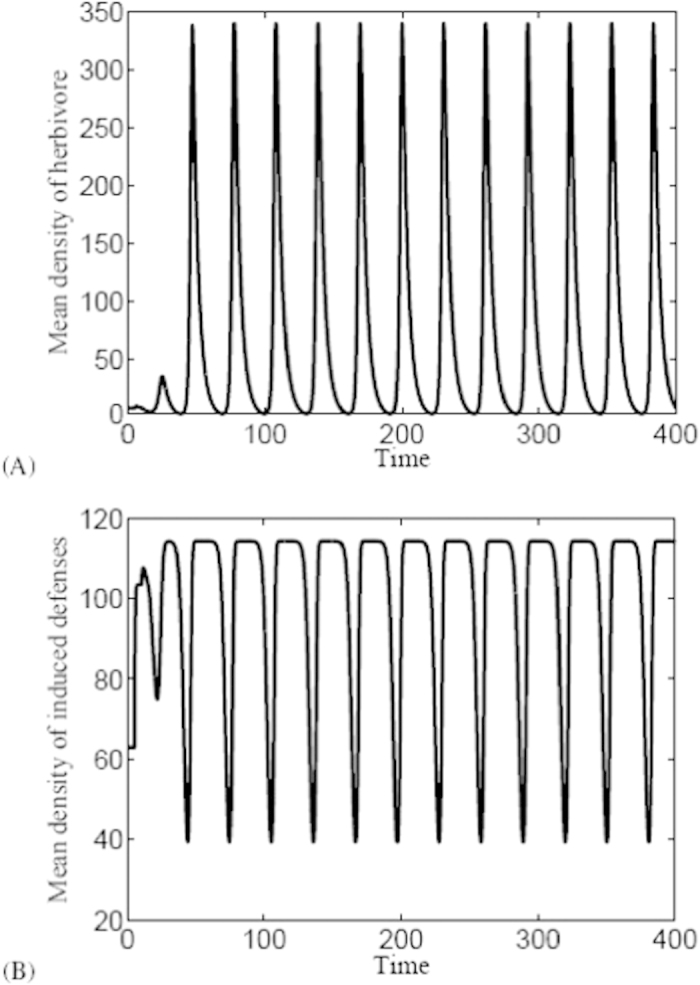
Synchrony of the herbivore and inducible defenses with *τ* = 8 and the other parameters are as the same in Table 1. This figure also shows that herbivore populations suffer a higher likelihood of extinction if time delay is too large.

Synchronization is a fundamental phenomenon arising in many biological contexts, which can be an important part of the function or malfunction of a biological system^33^. We checked that during the period of herbivore outbreak, herbivore populations and inducible defenses exhibit synchronous phenomenon (cf. Fig. 4). However, this figure also indicates that, when time delay is too large, the minimum value of herbivore density will reach zero which may cause the herbivore populations to run a high risk of extinction which can be seen from Fig. 4(A). This phenomenon can be explained in two different directions: on the one hand, the presence of synchronization may decrease the global persistence^34^,^35^,^36^; on the other hand, it was observed that herbivore can remain persistent when inducible defenses are small and thus it may go extinct with high density of inducible defenses^7^. In this sense, time delay plays a negative role for the persistence of herbivore populations.

As seen from Figs 2, 3, we know that herbivore populations have property of periodic solutions. One may ask whether the periodic solutions are stable or not. Based on stability analysis (see Method section), we obtained that for all the parameters sets to ensure the outbreak of herbivore, *β* < 0 which means the periodic solutions are stable. From biological point of view, time delay plays a positive role in herbivore persistence due to that it can keep the density of herbivore in a determined range from extinction.

**Figure 5:**
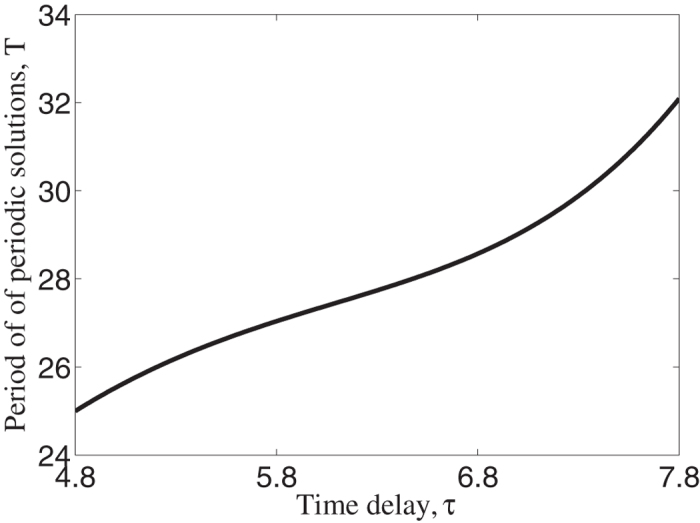
Period of periodic solutions with respect to time delay. As time delay increases, the period will increase with large amplitude.

The period of the periodic solutions has the following expression:


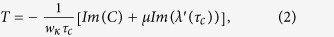


with 
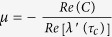
. Figure 5 shows the period of periodic solutions as a function of time delay. It shows that period is a increasing function of time delay. At the same time, the maximum value of herbivore density is becoming larger and the minimum value is becoming smaller as time delay increases.

### Combination of time delay and space promotes the growth of average density of herbivore populations

**Figure 6:**
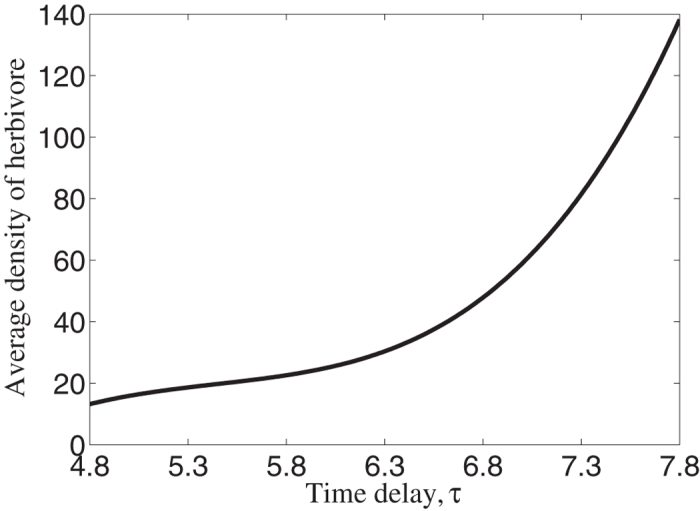
Average density of herbivore populations with respect to time delay. As time delay increases, average density increases and thus it suggests that time delay promotes the growth of average density of herbivore populations.

In the parameters sets which ensure the emergence of herbivore outbreak, it is found that average density of herbivore populations increases as time delay increases which was shown in Fig. 6. We checked that if herbivore populations do not outbreak, i.e., value of time delay is smaller than the critical value, this phenomenon can not be observed. Meanwhile, the results can not be obtained if space is not included. Accordingly, we concluded that interaction of time delay and space promotes the growth of average density of herbivore and then drive more resilience for herbivore populations. The existing results revealed that spatial scaling laws^37^, multiple scale spatial patterns^38^,^39^ or insects populations^40^ may increase the robustness in some biological systems. Therefore, our results enrich the findings in ecosystem functioning.

## Discussion

An extensive body of scientific research on inducible defenses of plant to herbivore populations demonstrated that inducible defenses may have great influences of dynamics of herbivore populations^41^,^42^,^43^,^44^. However, the studies on how inducible defenses exactly affect herbivore populations when space is under consideration, especially on the effect of herbivore outbreak, is still limited. As a result, a simple model to describe the interaction of inducible defenses and herbivore populations is investigated. It was found that time delay arising from plant defenses response to herbivore attacks can lead to periodic outbreak of herbivore populations. Furthermore, time delay has dual functions on the persistence of herbivore populations: large time delay may result in the extinction of herbivore and moderate value of time delay enlarges the possibility of herbivore persistence by keeping the periodic solutions to be stable. This finding implies that inducible defense with different intensities resolves the paradox of enrichment in the spatial sense.

Vos *et al.* found that inducible defenses play different roles on herbivore populations. On the one hand they promote local stability and thus persistence and on the other hand they may reduce the likelihood of herbivore persistence^13^. In our paper, we confirm the conclusions still hold when spatial effects are included. Meanwhile, we found that interaction of time delay and space may drive the resilience of herbivore populations on account of that time delay and space increase average density of herbivore populations during their outbreak period.

It should be noted that herbivore populations not only consume resources, but they are resources for other consumers. Consequently, they have much potential roles of connecting link between up and down trophic chains in evolution process of the whole ecosystems^45^. In this sense, it needs to be a balance in the control of herbivore populations and thus human beings can not blindly kill or protect herbivore populations.

## Method

### Mathematical Model

We give four main assumptions on our model: (1) To reflect delays in the deployment of inducible defenses, we assumed that induction changes at time *t* dependent on herbivore densities at *t* − *τ* time steps previously; (2) Inducible defenses is dependent on herbivore density (in saturation form) and the level of already inducible defenses; (3) In the absence of induced changes in plant quality, herbivore populations grows logistically with intrinsic rate *r* and carrying capacity *K*; (4) For some plants, their weeds can move in the space caused by environmental factors such as wind. Consequently, we consider that both inducible defenses and herbivore randomly move in the space with diffusion coefficients *d*_1_ and *d*_2_ respectively. Based on the above assumptions, we arrive at the following reaction diffusion equation:


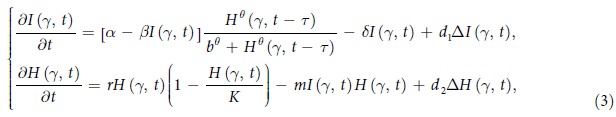


where *I*(*γ*,*t*) and *H*(*γ*,*t*) represent inducible defenses and herbivore density in both space and time. *α* is maximum per capita induced defenses, *β* is per unit reduction in the elicitation rate due to plant self-limitation, *δ* is per-unit induction decay rate, *m* is per unit reduction in the growth rate of herbivore caused by induction of defenses^32^, *γ* represents space, and Δ = ∂^2^/∂*x*^2^ is Laplacian operator in one-dimensional space. More details can be found in Table 1.

### Analysis on Critical Value

Mathematical speaking, if a system undergoes hopf bifurcation, then it will exhibit periodic solutions. For system (3), if it has hopf bifurcation, the herbivore populations will outbreak. In this sense, we need to find the critical value for herbivore outbreak.

Denote E^*^ = (I^*^,H^*^) as the positive equilibria of system (3). We deduce the eigenpolynomial associated with wavenumber *κ*:


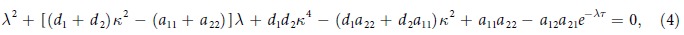


where 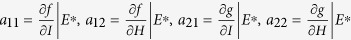 with 
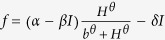
 and 
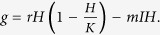


Setting *λ* = *iw*(*w* > 0) is a root of the eigenpolynomial (4) and separating the real and imaginary parts, one can have:
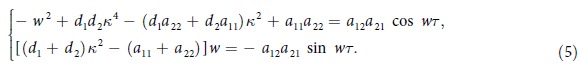


Then,


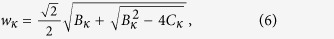


where 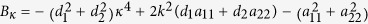 and 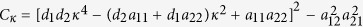. The corresponding critical value of time delay is:


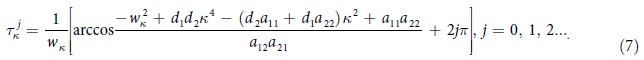


By calculations, the transversality condition 
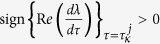
 holds. As a result, system (3) undergoes a spatial Hopf bifurcation at the equilibrium E^*^ = (I^*^,H^*^) when 
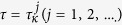
 and periodic solutions emerge in system (3) when 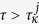.

### Stability of Periodic Solutions for Herbivore Populations

We can use normal form and the center manifold theory to investigate the stability of the bifurcated periodic solutions^49^. In order to determine the properties of Hopf bifurcating periodic solutions at the critical value, we can compute the following values:


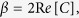


with 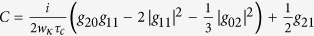. Since the expressions of *g*_02_, *g*_11_, *g*_20_ and *g*_21_ are complex, we omit them here. The bifurcating periodic solutions are stable (unstable) if *β* < 0 (*β* > 0).

## Additional Information

**How to cite this article**: Sun, G.-Q. *et al.* Effects of time delay and space on herbivore dynamics: linking inducible defenses of plants to herbivore outbreak. *Sci. Rep.* **5**, 11246; doi: 10.1038/srep11246 (2015).

## Figures and Tables

**Table 1:** Summary of the parameters used in system (3).

Parameter	Value	Comments	References
*α*	200	maximum induction rate per herbivore	47
*β*	1	per-unit reduction of induction rate by self-limitation	32
*δ*	0.75	per-unit induction decay rate	32
*b*	5	half-maximum for herbivore effectiveness of damage	47
*θ*	3	herbivore damage effectiveness shape tuning parameter	47
*r*	1	intrinsic rate of herbivore populations growth	32
*K*	10	herbivore carrying capacity	32
*m*	0.01	mortality rate by induction	32
*d*_1_	0.01	diffusion rate of plant populations	48
*d*_2_	0.01 ~ 1	diffusion rate of herbivore populations	49
